# Cyclodextrin–Epichlorohydrin–Cyanoguanidine Polymer for Resveratrol Delivery to Enhance Human Chondrocyte Function in Cartilage Repair

**DOI:** 10.1021/acs.biomac.5c02392

**Published:** 2026-04-22

**Authors:** Mahmoud A. Elmeligy, Forough Rasoulian, Shima Kalantarifard, Juraj Filo, Sahar Dinparvar, Ahmed M. Omer, Lucy Vojtová, Stefan Nehrer, Igor Lacík, Abolfazl Heydari

**Affiliations:** † Polymer Institute of the Slovak Academy of Sciences, Dúbravská cesta 9, 845 41 Bratislava, Slovakia; ‡ Center for Regenerative Medicine, University of Continuing Education Krems, 3500 Krems an der Donau, Austria; § Department of Organic Chemistry, Faculty of Natural Sciences, 164047 Comenius University, Ilkovičova 6, 842 15 Bratislava, Slovakia; ∥ Central European Institute of Technology, 613011 Brno University of Technology, 612 00 Brno, Czech Republic

## Abstract

Water-soluble β-cyclodextrin–epichlorohydrin polymers (CDPs) are widely used in drug delivery and regenerative medicine. Herein, we report a novel β-cyclodextrin–epichlorohydrin–cyanoguanidine polymer (CDPC) for resveratrol (RES) delivery in cartilage repair. Cyanoguanidine (CyG), a nitrogen-rich compound remaining nonprotonated at physiological pH, was incorporated at varying CyG/β-CD ratios to modulate the polymer properties. Structural characterization was performed by NMR, FT-IR, and CHN analyses. Compared with CDP, CDPC exhibited enhanced RES encapsulation that was attributed to additional intramolecular interactions. Dynamic light scattering revealed nanosized complexes (18 nm for CDPC/RES vs 4 nm for CDP/RES) with a near-neutral surface charge. CDPC showed intrinsic antioxidant activity, which was further enhanced upon RES loading. Both CDP and CDPC were cytocompatible and were efficiently internalized by human chondrocytes. Moreover, the CDP/RES and CDPC/RES systems improved the chondrocyte metabolic activity and extracellular matrix deposition, highlighting their potential as promising carriers for cartilage repair and regeneration.

## Graphical abstract

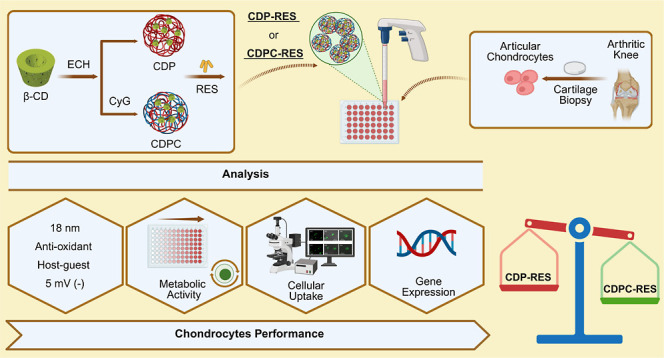

## 1 Introduction

Water-soluble β-cyclodextrin-epichlorohydrin polymers (CDP) have emerged as highly versatile materials, attracting considerable interest across diverse scientific fields including pharmaceutical research,^1–5^ biomedical engineering,^6^ wastewater treatment,^7^ and food science.^8,9^ These polymers are synthesized by chemical cross-linking of β-cyclodextrin (β-CD) using epichlorohydrin (ECH), a simple and widely used method that produces water-soluble CDPs.^3,10,11^ During the cross-linking process, the hydrophilic exterior of β-CD interacts with ECH by the hydroxyl groups of its sugar units, resulting in the formation of 2-hydroxypropyl ether (HPE) linkages, while the hydrophobic interior remains intact. This dual functionality, which integrates a polymeric network with the host–guest inclusion properties of β-CD, enables the efficient encapsulation of guest molecules. As a drug carrier, CDP improves drug solubility and enhances bioavailability, thereby contributing to increased therapeutic efficacy.^2,12^ However, the hydroxyl and ether functional groups present in CDPs, although beneficial for enhancing solubility, provide a limited capacity for forming hydrogen bonding and electrostatic interactions with drug molecules. This limitation reduces the drug encapsulation efficiency, particularly for therapeutics requiring robust intermolecular binding.

To address these challenges, the functionalization of CDPs with diverse molecules has been extensively studied to enhance drug–carrier interactions and optimize the performance of CDP-based delivery systems.^13^ For example, nitrogen-containing molecules, such as guanidine,^14^ primary amines,^15^ and quaternary ammonium salts,^16^ have demonstrated significant potential in addressing particularly the limited interaction capacity with target molecules. To further enhance the applicability of CDP-based systems in pharmaceutical applications, the investigation of alternative functional moieties has become a central focus within the CDP research community.

From this perspective, the present work focuses on the incorporation of cyanoguanidine (CyG) into CDP to create a new generation of functionalized CDP materials with potential applications across various fields, as outlined below. CyG is a nitrogen-rich chemical compound containing cyano (−C≡N) and guanidine (−NHC­(=NH)­NH_2_) functional groups, which enable CyG to participate in hydrogen bonding, dipole–dipole interactions, and, when protonated, electrostatic interactions.^17^ These features make it a suitable compound for various applications across agriculture, pharmaceuticals, and electronics involving CyG either in its raw form^18,19^ or as a precursor for synthesizing diverse products.^20–25^ CyG was used in the preparation of polymers,^23–25^ fertilizers,^18,24^ fire retardants,^22^ and adhesives.^20^ In medicinal and pharmaceutical areas, CyG primarily functions as a chemical intermediate rather than a direct therapeutic agent; however, its derivatives do exhibit biomedical potential. CyG groups enhance the bioactivity and therapeutic potential of sulfoximine derivatives.^26^ Metal complexes of CyG, particularly those with Cu­(II) and Ni­(II), show notable antimicrobial,^27,28^ DNA-binding,^27^ and antioxidant^21^ properties, aiding in combating infections and mitigating oxidative stress-related conditions. Additionally, CyG is used for targeted cancer therapies as a component of carbon nanodots with photothermal properties,^29^ and of polysaccharides, like chitosan, for enhancing the water solubility and antimicrobial efficacy.^30^ CyG-based polymers and composites are also used in the design of biomaterials for drug delivery and cell encapsulation.^23,25,31^ Given these advantages, the incorporation of CyG into the CDP structure offers a promising approach to enhance the properties of these polymers in multiple directions.

In this study, we investigate the potential application of the developed CDP as a drug delivery system for enhancing human chondrocyte function in cartilage repair. Effective drug delivery to chondrocytes remains a major challenge due to the dense, avascular, and negatively charged extracellular matrix (ECM) of cartilage, which hinders both penetration and retention of therapeutic agents.^32,33^ Thus, an efficient drug delivery system must facilitate targeted and sustained intracartilage delivery.^33,34^ Among the various approaches, the charge-based strategies using cationic carriers have demonstrated enhanced tissue affinity through electrostatic interactions with the negatively charged ECM.^35^ However, Vedadghavami et al. suggested that when hydrophobic interactions dominate within the synovial environment, this often leads to reduced cartilage accumulation, highlighting the importance of minimizing the carrier hydrophobicity for effective targeting.^36^ Moreover, the hydrogen bonding interactions were shown to synergize with electrostatic interactions that enhance both drug retention and delivery efficiency.^36^ These design principles are critical for developing platforms that achieve deep cartilage penetration and sustained therapeutic action. Beyond overcoming ECM-associated barriers, intracellular delivery provides an additional advantage by enabling direct drug delivery into chondrocytes.^37^ Importantly, uptake through nonendocytic pathways can prevent lysosomal degradation of carriers and their cargo, resulting in prolonged intracellular retention and improved therapeutic efficacy.^38^

Accordingly, in this work, CDPC was synthesized by cross-linking β-CD with ECH in the presence of CyG. CyG was selected for its nitrogen-rich and weakly basic structure, which remains neutral at physiological pH, while introducing functional groups capable of enhancing the hydrogen bonding through guanidine moieties and dipole–dipole interactions via cyano groups. The hypothesis is that CyG will enhance the drug encapsulation capacity of the polymers and facilitate interactions with cartilage components, thereby promoting targeted drug delivery to chondrocytes. The chemical structure of CDPC was confirmed by using NMR, FT-IR, and CHN elemental analysis. Resveratrol (RES), a hydrophobic polyphenol with anti-inflammatory,^39^ antioxidant,^40^ and chondroprotective properties,^41,42^ was selected as a therapeutic compound. By scavenging reactive oxygen species and suppressing pro-inflammatory and cartilage-degrading pathways, RES has emerged as a promising therapeutic candidate for cartilage repair.^42^ RES encapsulation studies were conducted to evaluate the complexation capabilities of both CDP and CDPC and to elucidate the effect of CyG incorporation on RES–polymer interactions, water solubility enhancement, and encapsulation efficiency. Complementary in vitro studies were conducted to evaluate the antioxidant activity of the polymers (CDP and CDPC) and of RES encapsulated in these polymers (CDP/RES and CDPC/RES complex). Finally, cytocompatibility, cellular uptake, and effects on cartilage matrix deposition in human chondrocytes were investigated to assess the potential of these polymers as functional drug carriers for cartilage repair applications.

## 2 Experimental Section

### 2.1 Materials

β-Cyclodextrin (β-CD) was purchased from Cyclolab R&D Ltd. (Budapest, Hungary). Epichlorohydrin (ECH, >99.0%) and resveratrol (RES, >99.0%) were obtained from TCI Europe N.V. Cyanoguanidine (CyG, 99%), potassium persulfate (K_2_S_2_O_8_, 99%), fluorescein 5(6)-isothiocyanate (FITC, ≥90%), sodium carbonate (Na_2_CO_3_, ≥99.5%), Trypsin–EDTA, phosphate-buffered saline (PBS, pH 7.2, and sterile-filtered), and paraformaldehyde (95%) were obtained from Sigma-Aldrich. Sodium hydroxide (NaOH, 99.59%), hydrochloric acid (HCl, 35%), and sodium chloride (NaCl, 99.98%) were obtained from Lach/ner. Ethanol (EtOH, 96%) was purchased from Mikrochem Trade, spol. s.r.o. Deuterium oxide (D_2_O, 99.9% D) was purchased from Eurisotop. 2,2′-Azino-bis­(3-ethylbenzothiazoline-6-sulfonic acid) diammonium salt (ABTS, 98%) was obtained from Thermo Scientific Chemicals. A dialysis tubing cellulose membrane with a molecular weight cutoff of 14,000 g·mol^–1^ was purchased from Sigma-Aldrich. Deionized water was used in the experiments.

### 2.2 CDP Synthesis

β-CD was cross-linked with ECH following the method reported in the literature^3^ with slight modifications. The amount of β-CD used in the feed ranged from 2 to 7 g. A representative example of the synthesis, with a feed of 2 g β-CD, is described in detail.

Two grams of β-CD (1.76 mmol) were dissolved in 6.4 mL of 33 wt % NaOH. Subsequently, 4.8 mL of ECH (61.22 mmol) was introduced to achieve an initial molar ratio [β-CD]_0_/[ECH]_0_ = 1:35 (subscript 0 refers to initial molar ratios of components). The reaction proceeded at 30 °C for 3 h with stirring at 600 rpm. The reaction was quenched with acetone, and the solvent was removed by decantation. The resulting solution was neutralized using 6 mol·L^–1^ HCl and transferred to dialysis tubing with a molecular weight cutoff of 14,000 g·mol^–1^. Dialysis against water was conducted while maintaining the pH of the water at around 7.5 for 3 days, with water exchanges performed at least three times daily. After dialysis, the content of the dialysis tubing was lyophilized to obtain CDP as a white powder.

### 2.3 CDPC Synthesis

β-CD was cross-linked with ECH in the presence of CyG at various molar ratios of [β-CD]_0_/[CyG]_0_ = 1:3, 1:5, and 1:10, using the constant molar ratio [β-CD]_0_/[ECH]_0_ = 1:35. The amount of β-CD used in the feed ranged from 2 to 7 g. A representative example of the synthesis, with a stoichiometric ratio of [β-CD]_0_/[ECH]_0_/[CyG]_0_ = 1:35:5 and a feed of 2 g of β-CD, is described in detail.

Two grams (1.76 mmol) of β-CD were dissolved in 6.4 mL of 33 wt % NaOH. To this solution 0.74 g (8.8 mmol) of CyG was added, followed by the addition of 4.8 mL (61.22 mmol) of ECH under continuous stirring. The reaction was carried out under the conditions described for the synthesis of CDP. After completion of the reaction, the brown powder product was purified and isolated using the purification protocol for CDP. The polymer yield was primarily determined as a gravimetric yield, defined as the ratio of the isolated polymer to the total initial weight of polymer-forming reagents (β-CD, ECH, and CyG), as expressed in eq 1. In addition, a β-CD-based yield, defined as the polymer-to-initial β-CD weight ratio, was calculated using eq 2:

1 $$\mathrm{Gravimetric}\;\mathrm{yield}\;\left(\%\right)=\frac{m_{\mathrm{polymer}}}{m_{\mathrm{polymer}‐\mathrm{forming}\;\mathrm{reagent}}}\times 100$$

2 $$\beta ‐CD‐based\;yield\;\left(\%\right)=\frac{m_{polymer}}{m_{\beta ‐CD}}\times 100$$

where *m*_polymer_ is the weight of the isolated dry polymer after lyophilization, and *m*_β‑CD_ is the weight of the initially added β-CD, and *m*_polymer‑forming reagent_ is the weight of the initially added β-CD, ECH, and CyG.

### 2.4 FITC-Labeling of Polymers

FITC-labeled CDP and CDPC-5 were synthesized following a previously reported method,^23^ with minor modifications. CDP (0.3 g) or CDPC (0.3 g) was dissolved in 15 mL of 0.1 mol·L^–1^ Na_2_CO_3_ (pH 11). Separately, FITC (15 mg, 0.039 mmol) was dissolved in 15 mL of the same buffer. The FITC solution was added dropwise to the polymer solution under continuous stirring. The reaction mixture was stirred at ambient temperature for 20 h in the dark. The reaction mixture was dialyzed against distilled water for 7 days using a dialysis membrane with a molecular weight cutoff of 14 000 g·mol^–1^ to remove unreacted FITC. The final products were obtained by freeze-drying and stored protected from light until further use.

### 2.5 Phase Solubility

The solubilization capacity of CDP and CDPC toward RES was determined according to the phase-solubility method of Higuchi and Connors.^43,44^ Phase solubility studies were performed in water at ambient temperature to investigate the effect of CDP and CDPC-5 on the solubility of RES. An excess amount of RES (15 mg) was added to 4 mL of water, followed by the addition of varying amounts of CDP and CDPC-5 to achieve their final concentrations ranging from 2.5 to 25 mg·mL^–1^. These suspensions were stirred for 72 h to reach equilibrium. The insoluble fraction was removed by filtration using nylon syringe filters with a 0.45 μm pore size. The RES content in the filtrates was quantified by UV–vis spectrophotometry at a wavelength of 310 nm. The resulting data were used to provide the phase solubility diagrams depicting the RES concentration complexed with a polymer in an aqueous environment alongside the CDP and CDPC concentrations, respectively. All experiments were conducted in triplicate.

### 2.6 RES Encapsulation Efficiency (EE) and Encapsulation Capacity (EC)

Polymer solutions were prepared at a concentration of 10 mg·mL^–1^ in water. Five milliliters of these solutions were mixed with the RES solution in EtOH at a concentration of 5 mg·mL^–1^. The weight ratio of CDP and CDPC to RES in the mixtures varied from 10:1 to 1:1. Each mixture was stirred at ambient temperature for 24 h to ensure equilibrium was reached, followed by the slow evaporation of solvents under air-drying conditions. Then, samples were washed thoroughly with EtOH three times to remove free RES, and the precipitate was separated by centrifugation at 6000 rpm for 15 min. The amount of free RES, which was washed with EtOH, was quantified by determining its concentration in EtOH using UV–vis spectroscopy at 310 nm. The drug encapsulation efficiency (EE) and drug encapsulation capacity (EC) were calculated as follows:

3 $$EE\left(\%\right)=\frac{m_{initial\;RES}-m_{free\;RES}}{m_{initial\;RES}}\times 100$$

where *m*_free RES_ (g) represents the weight of RES washed with EtOH, as measured by UV–vis spectrophotometry, and *m*_initial RES_ (g) is the weight of the initially added RES, and

4 $$EC\left(g/g\right)=\frac{m_{initial\;RES}-m_{free\;RES}}{m_{carrier}}$$

where *m*_free RES_ (g) represents the weight of RES washed with EtOH, as measured by UV–vis spectrophotometry, *m*_initial RES_ (g) is the weight of the initially added RES, and *m*_carrier_ is the weight of the CDP and CDPC-5 (g), respectively.

### 2.7 Polymer/RES Complex at a Weight Ratio of 1:1

A polymer/RES complex with a 1:1 weight ratio was prepared for further characterization and for in vitro studies. This complex was prepared using the method described in Section 2.5. In this case, after air-drying, the complex was dried in a vacuum oven at 40 °C overnight to ensure the complete removal of residual solvents.

### 2.8 Elemental Analysis

Elemental analysis was performed to measure carbon, nitrogen, and hydrogen contents using a Thermo Scientific Flash 2000 CHN Elemental Analyzer.

### 2.9 FT-IR Spectroscopy

FT-IR spectra were recorded by using a Bruker Tensor 27 FT-IR spectrophotometer. Samples were mixed with KBr and pressed into disks for analysis. Spectral scans were conducted over the range of 4000 to 400 cm^–1^.

### 2.10 NMR Analysis

NMR spectra were recorded on a Varian VNMRS 600 MHz spectrometer at 80 °C. The samples were introduced into 5 mm NMR tubes to which D_2_O was added. The tubes were kept at ambient temperature to dissolve the sample. The polymer concentrations were 20 mg·mL^–1^ for ^1^H NMR, ^13^C NMR, and ^1^H–^13^C heteronuclear single quantum coherence (HSQC) and 1 mg·mL^–1^ for diffusion-ordered spectroscopy (DOSY). For DOSY measurements of the polymer/RES complexes, a concentration of 10 mg·mL^–1^ was used. The proton chemical shifts expressed in parts per million were reported relative to the residual protonated solvent, HOD.

### 2.11 Molar Ratios of Components in Polymers

The molar content of the polymer components was determined and normalized relative to one mole of β-CD and is expressed as [β-CD]_p_/[HPE]_p_/[CyG]_p_ = 1/*x*/*y*, where *x* and *y* represent the number of HPE and CyG repeat units in polymers per β-CD units, respectively.

^1^H NMR was used to determine the molar ratio of β-CD to HPE. The proton assignments are described in Section 3.2 and illustrated in Figure 1. Specifically, the integration of the signal at 5.5–5.8 ppm corresponding to the anomeric protons (H1) of β-CD was compared with the integrated area in the 3.4–4.5 ppm region, which includes signals from the H2/H6 of β-CD as well as protons from HPE. The theoretical proton ratio of H1 to H2/H6 in β-CD is 7:42. Each repeating unit of poly­(HPE) contributes an additional 5 protons in the H2–H6 region. Based on this, the number of HPE units per β-CD was calculated using eq 5:

5 $$n_{HPE}=\frac{7\times \left(\frac{I_{CDH2-6+HPE}}{I_{CDH1}}\right)-42}{5}$$

where *I*_CDH1_ is the integrated signal for β-CD H1 protons (at 5.8 ppm), *I*_CDH2–6+HPE_ is the integrated signal for H2–H6 protons of β-CD and protons from HPE (3.4–4.5 ppm), 7 and 42 are the number of H1 and H2–H6 protons in β-CD, respectively, and 5 is the number of protons contributed by each poly­(HPE) repeating unit.

The content of CyG units was determined using elemental analysis (CHN) based on the nitrogen-to-carbon molar ratio, (N/C)_EA_, as derived from the weight ratio by conversion to molar ratio via atomic weights (multiplying by 12/14). The molar amount of CyG units per β-CD was calculated using eq 6:

6 $$n_{CyG}=\frac{{\left(N/C\right)}_{EA}\times \left(21+n_{HPE}\times 3\right)}{{nN}_{CyG}-{\left(N/C\right)}_{EA}\times {nC}_{CyG}}$$

where (N/C)_EA_ is the nitrogen-to-carbon molar ratio from CHN analysis, 21 is the number of carbon atoms in one β-CD, 3 is the number of carbon atoms per HPE, *n*_HPE_ is the number of HPE repeat units per β-CD, and *n*N_CyG_ and *n*C_CyG_ are the number of nitrogen and carbon atoms in one CyG unit, respectively.

### 2.12 Dynamic Light Scattering and Zeta Potential

Dynamic light scattering (DLS) and zeta potential measurements were performed at 25 °C using an Anton Paar Litesizer 500 instrument to evaluate the hydrodynamic diameter and zeta potential of the samples, respectively. Samples were prepared at concentrations of 5 and 10 mg·mL^–1^ in saline (0.9 wt % NaCl) at pH = 7. DLS and zeta potential measurements were conducted in triplicate with 10 and 150 runs per test, respectively.

### 2.13 UV–Vis Spectrophotometry

UV–vis spectra in the wavelength range of 200–800 nm were recorded using a Shimadzu UV-16 50PC UV–vis spectrophotometer.

### 2.14 Antioxidant Activity by the ABTS Assay

The antioxidant activity was assessed using a method involving scavenging of the stable synthetic radical cation ABTS^•+^, following established protocols.^45^ ABTS^•+^ was generated by combining equal volumes of 7 mmol·L^–1^ ABTS solution in water and 2.45 mmol·L^–1^ K_2_S_2_O_8_ solution in water. This mixture was then incubated in the dark at ambient temperature overnight to allow the complete formation of ABTS^•+^. Subsequently, 1 mL of ABTS^•+^ solution was diluted with water until its absorbance reached approximately 1.0 at 734 nm by using a UV–vis spectrophotometer. Then, 2 mL of ABTS^•+^ solution was mixed with 50 μL of polymer solutions in water to reach a final concentration of 0.12, 0.24, and 0.48 mg·mL^–1^ of polymers, as well as 0.12 mg·mL^–1^ of the polymer/RES complex. The absorbance was measured in triplicate after a 15 min incubation period at a wavelength of 734 nm. ABTS^•+^ scavenging was calculated using eq 7:

7 $${ABTS}^{\cdot +}\;scavenging\;activity\;\left(\%\right)=\frac{{Abs}_{control}-{Abs}_{sample}}{{Abs}_{control}}\times 100$$

where Abs_control_ is the absorbance of the ABTS^•+^ solution mixed with the same volume of the solvent used for testing the samples, and Abs_sample_ is the absorbance of the ABTS^•+^ solution mixed with the test sample.

### 2.15 Cellular Performance

#### 2.15.1 Isolating and Growing Human Chondrocytes

Human articular cartilage was taken from patients diagnosed with osteoarthritis and undergoing complete knee arthroplasty at the University Hospital Krems. Before the study, the regional ethics committee approved this study (GS1-EK-4/761-2021) and all patients gave informed consent. Cartilage and bone specimens were transported to the research facility in a sterile container in phosphate-buffered saline (PBS) with antibiotics (penicillin 200 U·mL^–1^; streptomycin 0.2 mg·mL^–1^; Sigma-Aldrich).

To isolate human chondrocytes, articular cartilage was chopped into 2 mm^3^ pieces and digested with Liberase TM (0.2 WU/mL, Roche Diagnostics GmbH) in GIBCO DMEM/F12 GlutaMAX-I (Invitrogen, LifeTech Austria) with antibiotics and Amphotericin B. The digestion took between 18 and 22 h at 37 °C with continuous agitation. Undigested material was removed from the chondrocyte suspension by filtering it through a 40 μm Cell Strainer (BD, Franklin Lakes, USA). The cells were washed, centrifuged (10 min, 500 *× g*, room temperature), and resuspended in a growth medium with antibiotics, Amphotericin B, 10% FCS (GIBCO by Life Technologies), and 0.05 mg·mL^–1^ Vitamin C (Sigma-Aldrich). After isolation, chondrocytes were seeded into 75 cm^2^ culture flasks at a density of 1 × 10^4^ cells·cm^–2^ for cell expansion and cultivated at 37 °C in a humidified atmosphere with 5% CO_2_. The medium was refreshed every 2–3 days until reaching about 80% confluency. Accutase (1.5 mL/flask; Sigma-Aldrich) was used for chondrocyte detachment. Chondrocytes from three different donors were used.

**Figure 1:**
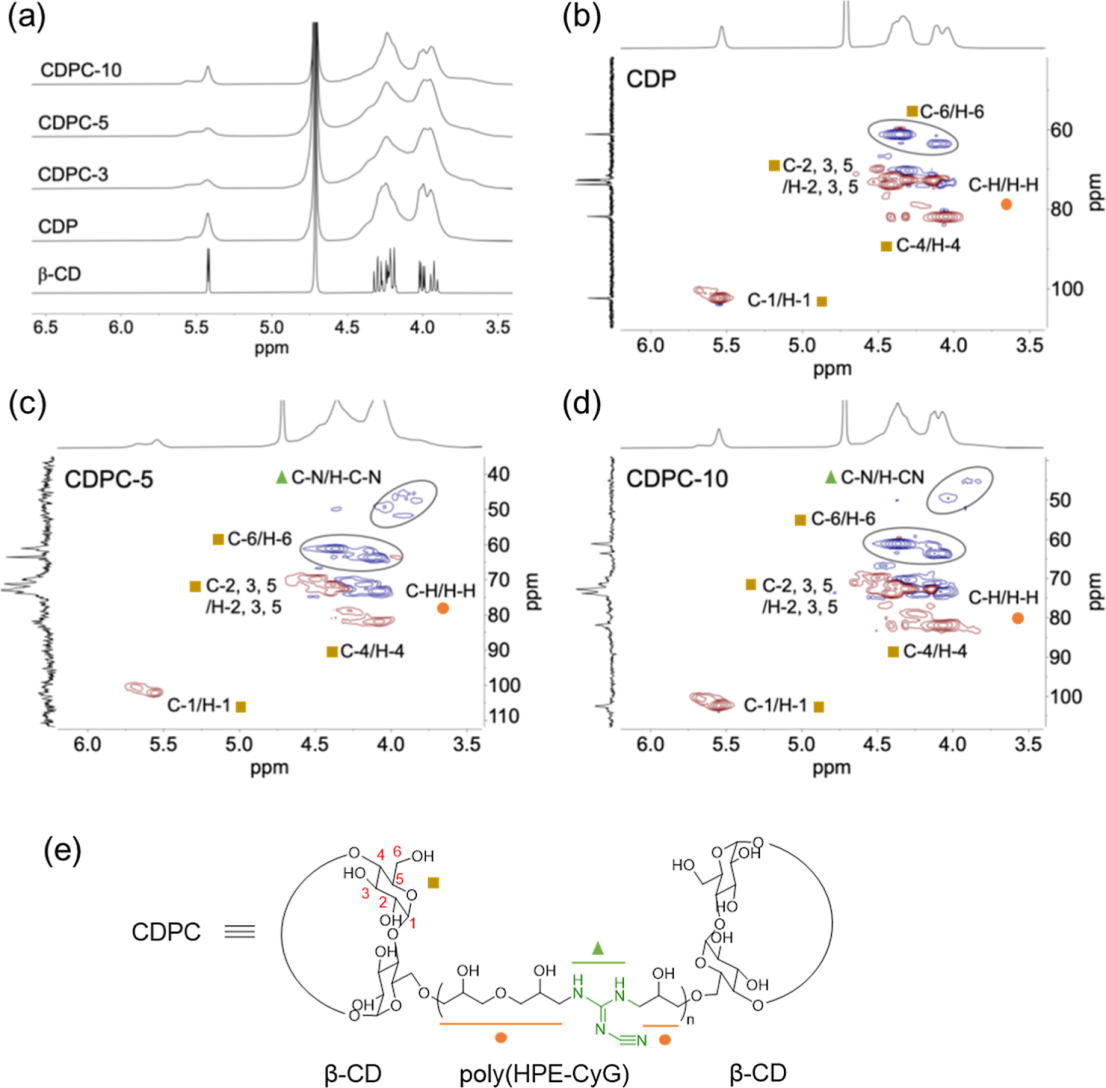
(a) ^1^H NMR (D_2_O, 600 MHz, 80 °C) of β-CD, CDP, CDPC-3, -5, and -10. ^1^H–^13^C HSQC spectra (D_2_O, 600 MHz, 80 °C) of (b) CDP, (c) CDPC-5, and (d) CDPC-10. (e) The chemical structure of CDPC is provided to assist in the spectra interpretation.

**Scheme 1:**
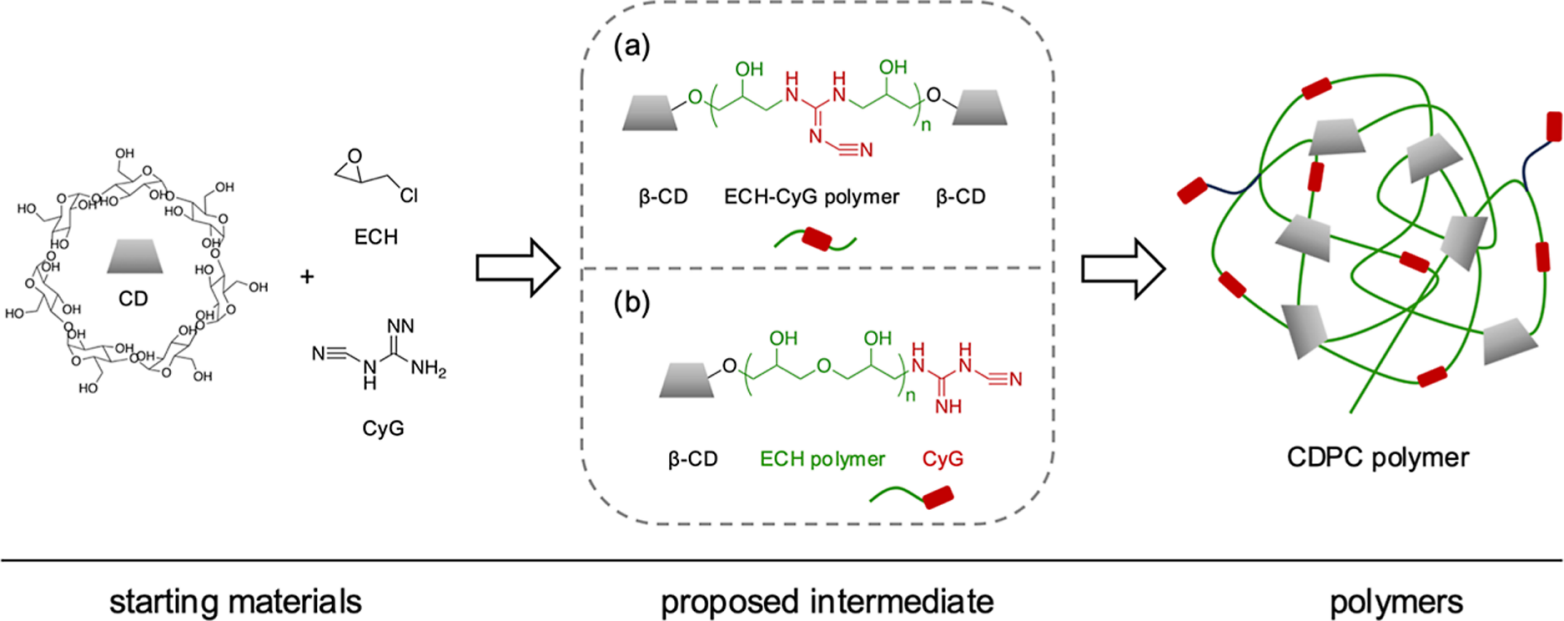
Schematic Representation of the Reaction of β-CD and CyG in the Presence of ECH toward the Formation of CDPC; (a,b) Illustrate Two Proposed Formation Mechanisms and Representative Intermediates in the Condensation of β-CD, ECH, and CyG. (a) CyG Incorporated within the Polymer Backbone and (b) CyG Positioned at the Terminal End

#### 2.15.2 Metabolic Activity of Chondrocytes by the XTT Assay

The metabolic activity of chondrocytes derived from a single donor was assessed. Detached chondrocytes were seeded in 96-well plates at a density of 5 × 10^3^ cells·cm^–2^ and subsequently exposed to solutions of polymers and polymer/RES complexes in serum-free growth media (treated cells) at five different concentrations, ranging from 0.125 to 10 mg·mL^–1^. After 24, 48, and 72 h, the metabolic activity of chondrocytes was measured by the XTT assay (Cell Proliferation Kit II, Roche Diagnostics, Switzerland). 50 μL of the XTT reagent prepared according to the manufacturer’s protocol was added to each well, followed by incubation for 4 h at 37 °C in a 5% CO_2_ environment. A multimode microplate reader (Synergy 2, Winooski, USA) with Gen 5 software was used to measure absorbance at 492 nm, with 690 nm as a reference wavelength for background correction, in accordance with the XTT assay protocol. The medium for chondrocytes with an XTT reagent was used as a control for each measurement. The metabolic activity of chondrocytes in the presence of polymers was determined by normalizing the absorbance relative to the control (chondrocytes cultured in the absence of polymers) according to eq 8

8 $$metabolic\;activity=\frac{{Abs}_{treated\;cells}}{{Abs}_{control}}$$

where Abs_treated cells_ is the absorbance of treated cells at 492 nm and Abs_control_ is the absorbance of untreated (control) cells at 492 nm.

#### 2.15.3 Polymer Uptake with Chondrocytes

Human chondrocytes (5 × 10^5^ cryopreserved cells, Sigma-Aldrich) were thawed and cultured in a Chondrocyte Growth Medium (ready-to-use kit containing Basal Medium and Supplement Mix, 500 mL; Sigma-Aldrich). Chondrocytes were seeded into T-75 culture flasks at an initial density of 5 × 10^5^ cells·mL^–1^ and maintained at 37 °C in a humidified incubator with 5% CO_2_ until reaching approximately 80% confluency. Upon confluency, cells were washed with PBS and detached using Trypsin–EDTA. The resulting cell suspension was centrifuged, resuspended in a fresh complete medium, and counted prior to further experimental use.

For polymer uptake experiments, 2 × 10^5^ cells were seeded into glass-bottom Petri dishes in 500 μL of the complete medium and incubated overnight at 37 °C and 5% CO_2_ to allow cell attachment. Following incubation, cells were treated with FITC-labeled CDP and CDPC-5 at a concentration of 0.125 mg·mL^–1^ for 3 h. After treatment, cells were washed three times with PBS to remove unbound polymers, fixed with 4% paraformaldehyde for 15 min at ambient temperature, and washed again with PBS.

A confocal laser scanning microscopy (CLSM) microscope Zeiss LSM 980 (Carl Zeiss Microscopy GmbH, Jena, Germany) equipped with an Airyscan 2 detector and a 40×/1.2 NA Apochromat objective was used to evaluate the uptake of FITC-fluorescently labeled polymers with chondrocytes. Both FITC fluorescence and transmitted-light channels were acquired, and Z-stack images were collected to assess the 3D intracellular localization of polymers.

#### 2.15.4 Cartilage Matrix Production with RNA Extraction and the Quantitative Real-Time Polymerase Chain Reaction (qRT-PCR) of Chondrocytes

Chondrocytes from two different donors, labeled as donor I and donor II, were seeded in 6-well plates at a density of 2 × 10^5^ cells·cm^–2^. Gene expression analysis was conducted at 1- and 3 day post-treatment. RNA was isolated from chondrocytes treated with 0.125 mg·mL^–1^ CDP/RES and CDPC-5/RES using the High Pure RNA Isolation Kit (Roche Diagnostics, Basel, Switzerland), with adjustments for direct extraction from 6-well plates, with untreated chondrocytes used as a control. Specifically, 400 μL of lysis buffer and 200 μL of PBS were added to each well and shaken for 30 s. The lysate was transferred to a high-purity RNA spin column. The remaining steps followed the manufacturer’s protocol. cDNA was synthesized from mRNA using the Transcriptor First Strand cDNA Synthesis Kit (Roche Diagnostics), with MS2 RNA added for stabilization. Quantitative PCR (qPCR) was performed in triplicate using a LightCycler 96 and FastStart Essential DNA Probe Master (Roche Diagnostics). Primer pairs (Table 2) for the cartilage-specific genes (COL2A1, COL1A1, ACAN, SOX9) and catabolic genes (MMP3, MMP13) were designed using IDT software and synthesized by IDT (Leuven, Belgium). Glyceraldehyde-3-phosphate dehydrogenase (GAPDH) is used as the housekeeping gene. The relative expression was calculated using the 2^–ΔCt^ method, comparing target gene expression to the reference gene.^46^

**Table 1:** Reaction Conditions for the Synthesis of CDP and CDPC^a^ ^,^ ^b^

sample code (*n*)	[β-CD]_0_/[CyG]0	gravimetric yield (%)	N/C weight ratio	[β-CD]_p_/[HPE]_p_/[CyG]_p_
CDP (3)	1:0	22 ± 3	0	1:6:0
CDPC-3 (3)	1:3	17 ± 3	0.089 ± 0.004	1:9:1.3
CDPC-5 (4)	1:5	15 ± 3	0.124 ± 0.007	1:10:2
CDPC-10 (3)	1:10	14 ± 4	0.159 ± 0.010	1:10:2.6

a *n* is the number of repeats of individual synthesis. The subscripts 0 and p refer to the initial molar ratios of components and their corresponding proportions in the final polymer composition, respectively. Standard deviation for the HPE content in both CDP and CDPC polymers, expressed as [β-CD]_p_/[HPE]_p_/[CyG]_p_, is less than ±0.5. Molar ratios of components in polymers are calculated in Section 2.11.

b The molar ratio of [β-CD]_0_/[ECH]_0_ equals to 1:35. The reaction proceeded using 28 wt % of the β-CD solution in 33 wt % NaOH at 30 °C for 3 h.

**Table 2:** Sequences of Primers and Conditions Used in RT-qPCR

target gene	primer forward	primer reverse
GAPDH	CTCTGCTCCTCCTGTTCGAC	ACGACCAAATCCGTTGACTC
COL2A1	GTGTCAGGGCCAGGATGT	TCCCAGTGTCACAGACACAGAT
ACAN	CCTCCCCTTCACGTGTAAAA	GCTCCGCTTCTGTAGTCTGC
SOX9	TACCCGCACTTGCACAAC	TCTCGCTCTCGTTCAGAAGTC
COL1A1	GGGATTCCCTGGACCTAAAG	GGAACACCTCGCTCTCCAG
MMP13	TTTCCTCCTGGGCCAAAT	GCAACAAGAAACAAGTTGTAGCC
MMP3	CAAAACATATTTCTTTGTAGAGGACAA	TTCAGETATTCGCTTGGGAAA

### 2.16 Statistical Analysis

A two-way ANOVA with Tukey’s test was performed for the statistical analysis in GraphPad Prism 10 (GraphPad Software Inc.), with a significance threshold of *p < 0.05. Means ± standard deviations (SD) were used to report the findings of the experiments, which were carried out in triplicate.

## 3 Results and Discussion

The polymerization of β-CD using ECH in the presence of CyG proceeds via a polycondensation reaction in an alkaline medium. This process follows a sequential, multistep mechanism involving the stepwise formation of polymer chains through the condensation of β-CD, ECH, and CyG. The mechanism of CDP formation was described by Renard et al.,^10^ where, under alkaline conditions, the alkoxide sites of β-CD react with the epoxide ring of ECH, leading to the formation of HPE linkages, resulting in glyceryl bridges between β-CD units. Simultaneously, ECH can undergo self-polymerization, which results in the formation of poly­(HPE) that interconnects multiple β-CD units.

Scheme 1 shows the proposed formation mechanism of CDPC with the introduction of CyG into the β-CD–ECH reaction system. Two principal reaction pathways, resulting in two distinct intermediates, are conceptually proposed. In the first pathway (Scheme 1a), the amino groups of CyG react with the epoxide groups of ECH and subsequently polymerize with additional ECH molecules, forming the poly­(HPE-CyG) chain, which links two β-CD molecules via reactions with their deprotonated hydroxyl (alkoxide) groups. In the second pathway (Scheme 1b), the alkoxide groups of β-CD and the amino groups of CyG react with the epoxide ring of ECH, leading to the formation of glyceryl bridges that interconnect β-CD and CyG. Ultimately, this polymerization process results in the formation of a cross-linked CDPC in which β-CD and CyG are interconnected through HPE. CyG units can be incorporated at different positions within the polymer network. The reaction proceeds via both mid-chain (Scheme 1a) and end-chain (Scheme 1b) incorporation of CyG, resulting in a polymer composed of networks containing CyG units either embedded within the polymer backbone or located at the terminal positions.

The reaction products, as outlined in Table 1, were evaluated gravimetrically by measuring the polymer weight retained in dialysis tubing with a molecular weight cutoff of 14,000 g·mol^–1^. The gravimetric yields were approximately 22% for CDP and 14–17% for CDPC variants. The apparently low yields result from including ECH added in large excess (β-CD/ECH = 1:35) in the calculation, whereas the actual ECH incorporation in the polymers was much lower (1:6 for CDP and ∼1:10 for CDPC). The slightly lower yield observed for CDPCs is likely due to reduced cross-linking efficiency resulting from CyG incorporation during polymerization. Nevertheless, all CDPC formulations exhibited comparable gravimetric yields, indicating similar overall polymer recovery, despite differences in CyG content. In parallel, the recovered polymer mass was normalized to the initial β-CD mass to determine the β-CD-based yield, yielding approximately 85% for CDP and around 65% for the CDPCs. The SD value for a β-CD-based yield of ±10%, derived from individual syntheses, was considered within an acceptable range.

The chemical composition of the synthesized polymers was tailored by varying the initial molar feed ratios of the reactants. The carbon-to-nitrogen weight ratio results (CHN elemental analysis), presented in Table 1, indicate the presence of nitrogen, implying the incorporation of CyG into the polymer. While the initial molar ratio of [β-CD]_0_/[CyG]_0_ varied from 1:0 to 1:10, the corresponding incorporation of CyG in the final polymers, represented by [CyG]_p_, increased progressively but not linearly, reaching values of 0, 1.3, 2.0, and 2.6, respectively. This suggests that while the CyG incorporation is dose-dependent, its efficiency decreases at higher feed ratios, likely due to steric hindrance or limited availability of reactive sites. Interestingly, the number of HPE units per β-CD also increased alongside CyG incorporation, from 6 to 10, indicating that the presence of CyG may influence the extent of cross-linking or branching via ECH.

### 3.1 FT-IR Spectroscopy

Figure S1 presents the FTIR spectra of CDP and CDPC-3, -5, and -10. In the FTIR spectrum of CDP, characteristic bands are observed, including a dominant O–H stretching vibration in the range of 3400–3300 cm^–1^, a C–H stretching vibration between 2950–2900 cm^–1^, a C–C stretching vibration at 1642 cm^–1^, and a C–O stretching vibration between 1152–1030 cm^–1^, corresponding to the β-CD and glycerol units from the HPE. Notably, the appearance of a signal at 2172 cm^–1^ in the spectra of the CDPC polymers indicates the presence of the cyano (−C≡N). In addition, the characteristic bands observed at approximately 1640–1670 cm^–1^ correspond to guanidine (−C=N–H) groups. This confirms the incorporation of CyG into the CDPC polymer structure, as indicated by the CHN analysis.

### 3.2 NMR

The ^1^H NMR spectra of β-CD, CDP, and CDPC-3, -5, and -10 are shown in Figure 1. A comparison between β-CD and the polymer samples (CDP and CDPC) reveals the presence of broader peaks in the 3.7–4.5 ppm region, suggesting structural modifications of β-CD upon polymerization. Additionally, signals corresponding to the HPE also appear in this region, overlapping with the signals of β-CD, as reported in the literature.^9^ Furthermore, in addition to the signal observed at 5.5 ppm, a new peak at 5.8 ppm emerges in both CDP and CDPC samples that is attributed to the anomeric proton of a modified β-CD monomer unit, further confirming the structural modification of β-CD. However, due to the complex nature of CDP and CDPC, the structural information obtained from ^1^H NMR spectra is limited. While CHN analysis and FT-IR spectroscopy provide evidence for the presence of CyG, the ^1^H NMR spectra do not exhibit distinct signals, confirming the CDPC structure. Therefore, to gain more detailed structural insights into the presence of CyG in CDPC by NMR, ^1^H–^13^C HSQC spectroscopy was performed.

The HSQC spectra of CDP, CDPC-5, and CDPC-10 are shown in Figure 1. For the β-CD moiety, the anomeric carbons (C-1) typically resonate in the region of 5.5–5.8 ppm (^1^H) and 100–105 ppm (^13^C), while secondary hydroxyl carbons (C-2, C-3, C-4, and C-5) and primary hydroxyl carbons (C-6) appear at 5.0–4.5 ppm (^1^H) with corresponding carbon signals at 60–85 ppm (^13^C). The incorporation of HPE as a cross-linker introduces additional aliphatic carbon signals in the regions of 4.0–4.3 ppm (^1^H) and 70–75 ppm (^13^C). The proton and carbon chemical shifts of CDP are in agreement with the reported values.^5^ In the HSQC spectra of CDPC compared to CDP, distinct cross-peaks at 3.8 and 4.0 ppm and their corresponding carbon signals at 45–55 ppm confirm the presence of C–N/H–C–N bonds, providing direct evidence for the incorporation of CyG into the polymer structure through amine linkages.

### 3.3 DOSY

DOSY is a widely utilized technique for mixture analysis^47^ and is particularly valuable for assessing the purity of polymeric samples, primarily in relation to their molecular weight distribution.^48^ This method enables the separation of chemical species based on their diffusion coefficients. The DOSY data for β-CD, CDP, and CDPC-5 are presented in Figure 2, expressing the diffusion coefficients (*D*) in cm^2^·sec^–1^ corresponding to the ^1^H chemical shifts of the samples, as described in Section 3.2. In all spectra, the diffusion coefficient of the HOD is equal to 2.2 × 10^–5^ cm^2^·sec^–1^. The diffusion coefficient of β-CD in D_2_O is 2.9 × 10^–6^ cm^2^·sec^–1^, while both CDP and CDPC-5 exhibit a lower diffusion coefficient of 7.5 × 10^–7^ cm^2^·sec^–1^. Notably, the DOSY spectra of CDP and CDPC-5 show no detectable signal corresponding to the diffusion coefficient of β-CD, confirming the effectiveness of the purification protocol. Furthermore, the presence of a singular diffusion signal in the DOSY spectra of CDP and CDPC indicates that these polymers are of high purity and uniformity in terms of the molecular weight distribution.

**Figure 2:**
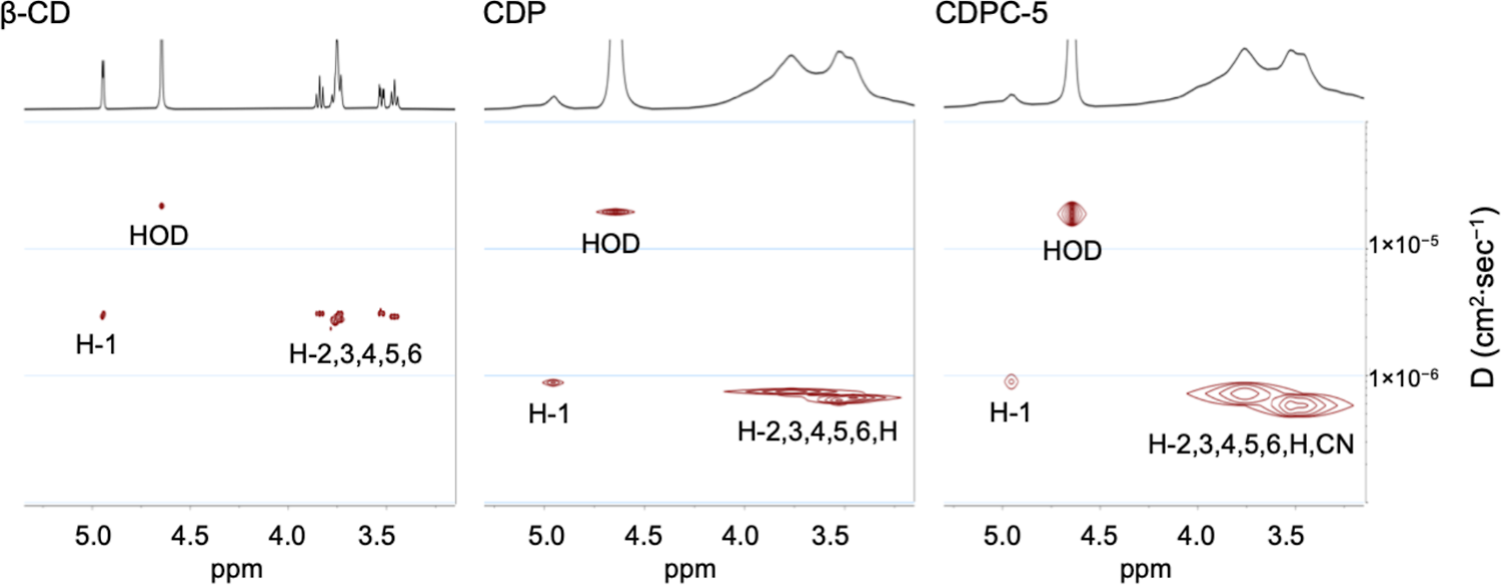
DOSY spectra (D_2_O, 600 MHz, 25 °C) of β-CD, CDP, and CDPC-5. The *x*-axis represents the ^1^H chemical shift of the samples in D_2_O, while the *y*-axis corresponds to the diffusion dimension. The ^1^H signal assignments are included in the spectra.

### 3.4 Potential of Polymers to Encapsulate RES

#### 3.4.1 Phase Solubility

RES exhibits a poor aqueous solubility^49^ of only ∼40 μg·mL^–1^ and low bioavailability both in humans^50^ and in animal models,^51^ which limits its clinical applicability. β-CD-based encapsulation has been developed as an effective method to enhance the aqueous solubility of RES.^52^ In particular, β-CD derivatives, such as hydroxypropyl-β-CD,^40,53,54^ randomly methylated-β-CD,^54^ sulfobutyl ether-β-CD,^55^ and CDP,^4^ have demonstrated a substantially enhanced RES solubility through the formation of inclusion complexes within their hydrophobic cavities.

The solubilization capacity of CDs is commonly evaluated using the phase-solubility method.^44^ In this study, we investigated the solubilizing efficiency of CDP and CDPC-5 for RES by using this approach. The resulting phase-solubility diagrams, presented in Figure 3a, show the relationship between RES solubility and varying concentrations of CDP and CDPC-5. The slope of the phase-solubility linear fits is indicative of the solubilization potential of the CDs.^44^ For CDP, the slope was approximately 0.14, whereas CDPC-5 exhibited a steeper slope of 0.20. Linear regression analysis demonstrated a significantly stronger concentration-dependent increase in RES concentration for CDPC-5 compared to CDP (*p* < 0.05), indicating an enhanced complexation ability and a greater increase in RES solubility. This performance of CDPC is attributed to the presence of CyG moieties, which act as external hydrophilic groups attached to the β-CD cavity and promote the inclusion complex formation via hydrogen bonding interactions with the hydroxyl groups of RES, thereby enhancing the efficiency of complexation. Similar improvements in RES complexation efficiency through β-CD functionalization with an external hydrophilic moiety have been reported in previous studies comparing β-CD and hydroxypropyl-β-CD,^40^ as well as β-CD and CDP.^4^ To further assess the influence of CyG content, CDPC-3 and CDPC-10 were evaluated at a polymer concentration of 18 mg·mL^–1^. At this concentration, no statistically significant differences were observed among the CDPC variants, while all CDPC formulations exhibited a statistically significant improvement in RES solubility compared to CDP. Notably, RES solubility was insensitive to the CyG content within the CDPC structure over the range of initial CyG concentrations used for CDPC synthesis. This suggests that the solubilization process is dominated by β-CD inclusion complexation, while CyG plays an additional facilitating contribution.

**Figure 3:**
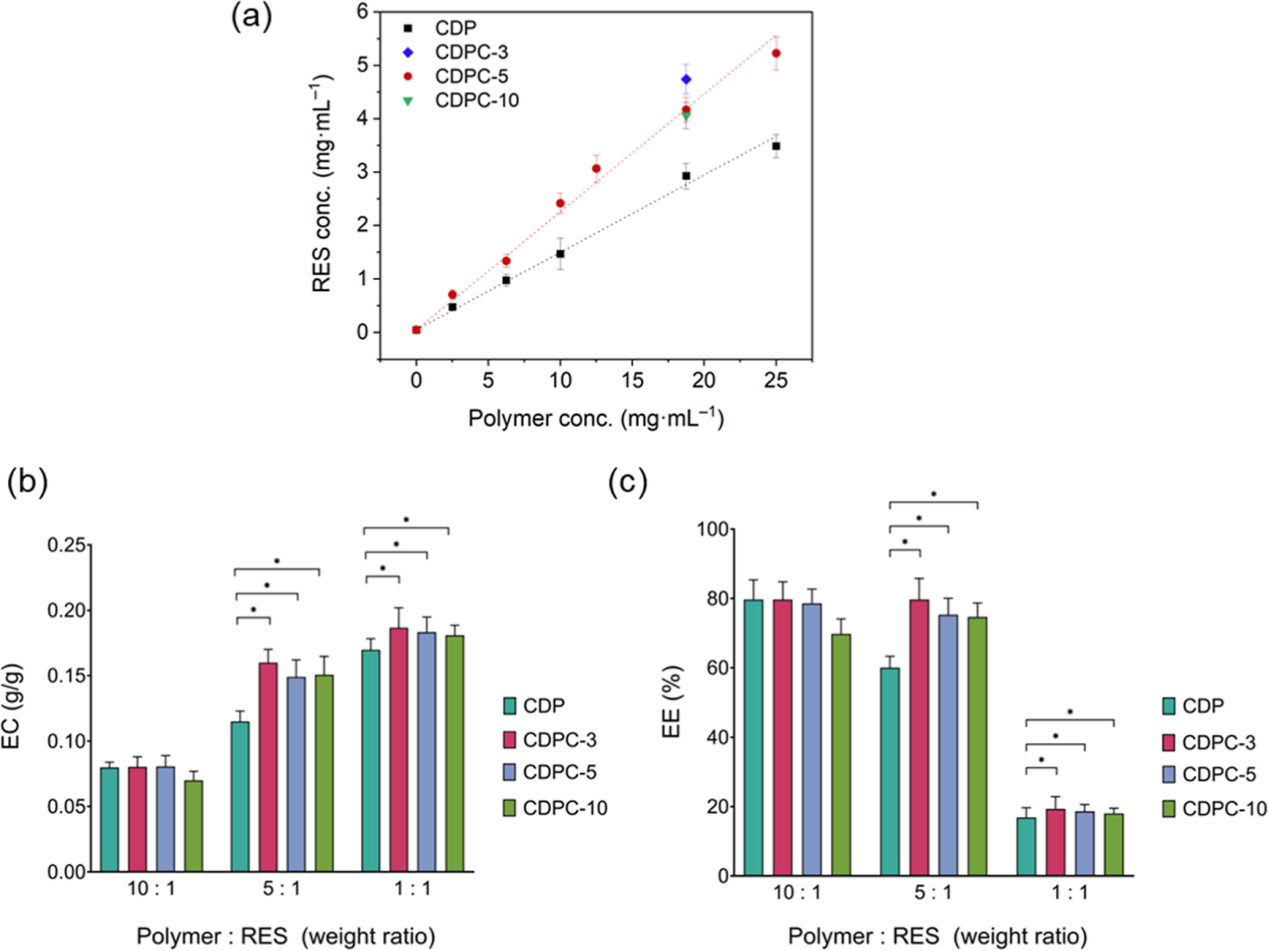
(a) Phase-solubility linear fits depicting the amount of RES complexed with polymers CDP and CDPC-5, in an aqueous environment as a function of polymer concentration. For comparison, RES complexation by CDPC-1 and CDPC-10 is shown at a polymer concentration of 18 mg·mL^–1^. A value of *p* < 0.05 is considered statistically significant. (b) Encapsulation efficiency (EE) and (c) encapsulation capacity (EC) of RES by CDP and CDPC (CDPC-3, -5, and -10) at polymer-to-RES weight ratios of 10:1, 5:1, and 1:1. Statistical significance is indicated by *p < 0.05, **p < 0.01, ***p < 0.001, ****p < 0.0001.

#### 3.4.2 RES Encapsulation Efficiency (EE) and Encapsulation Capacity (EC)

Figure 3b,c presents the encapsulation efficiency (EE, %) and encapsulation capacity (EC, g/g) of RES encapsulated by CDP and CDPC polymers containing varying CyG contents (CDPC-3, CDPC-5, and CDPC-10) at the polymer-to-RES weight ratios of 10:1, 5:1, and 1:1. The data reveal that EE increases with a higher polymer-to-RES ratio, whereas EC exhibits a decreasing trend in the same direction. At the 5:1 and 1:1 ratios, all CDPC formulations showed significantly higher EE values compared to CDP (*p* ≤ 0.05), while no statistically significant differences were observed among CDPC-3, CDPC-5, and CDPC-10. However, at the 10:1 ratio, EE did not differ significantly among the formulations. For EC, significant increases were observed for all CDPC groups compared to CDP at both the 5:1 and 1:1 ratios (*p* ≤ 0.05), with the 1:1 ratio yielding the highest EC values overall. The absence of significant differences at the 10:1 ratio is likely due to the relatively low amount of RES, which allows nearly complete encapsulation by the available polymer. Overall, these findings indicate that CyG incorporation enhances the encapsulation performance of CDPC compared with CDP, consistent with the trends observed in the phase-solubility studies. Based on these findings, a polymer-to-RES weight ratio of 1:1 was selected for complex preparation, as this ratio exhibited the highest EC, indicating the most effective conditions for RES encapsulation by the polymer.

### 3.5 Characterization of the Polymer/RES Complex

#### 3.5.1 NMR

Figure 4a,b shows the ^1^H NMR spectra of RES, CDP/RES, and CDPC-5/RES complexes, with the spectrum of CDP included for comparison. The chemical shifts of RES protons are highlighted. Proton signals corresponding to RES appear in the spectra of both CDP/RES and CDPC-5/RES, confirming the presence of RES and indicating the complexation of RES by these polymers. The complexes were washed with ethanol to remove nonencapsulated RES, further supporting that the observed signals arise from the polymer-encapsulated RES. Notably, slight variations in the chemical shifts of the RES proton signals are observed after complexation compared to free RES, suggesting the interaction between RES and the polymer.

**Figure 4:**
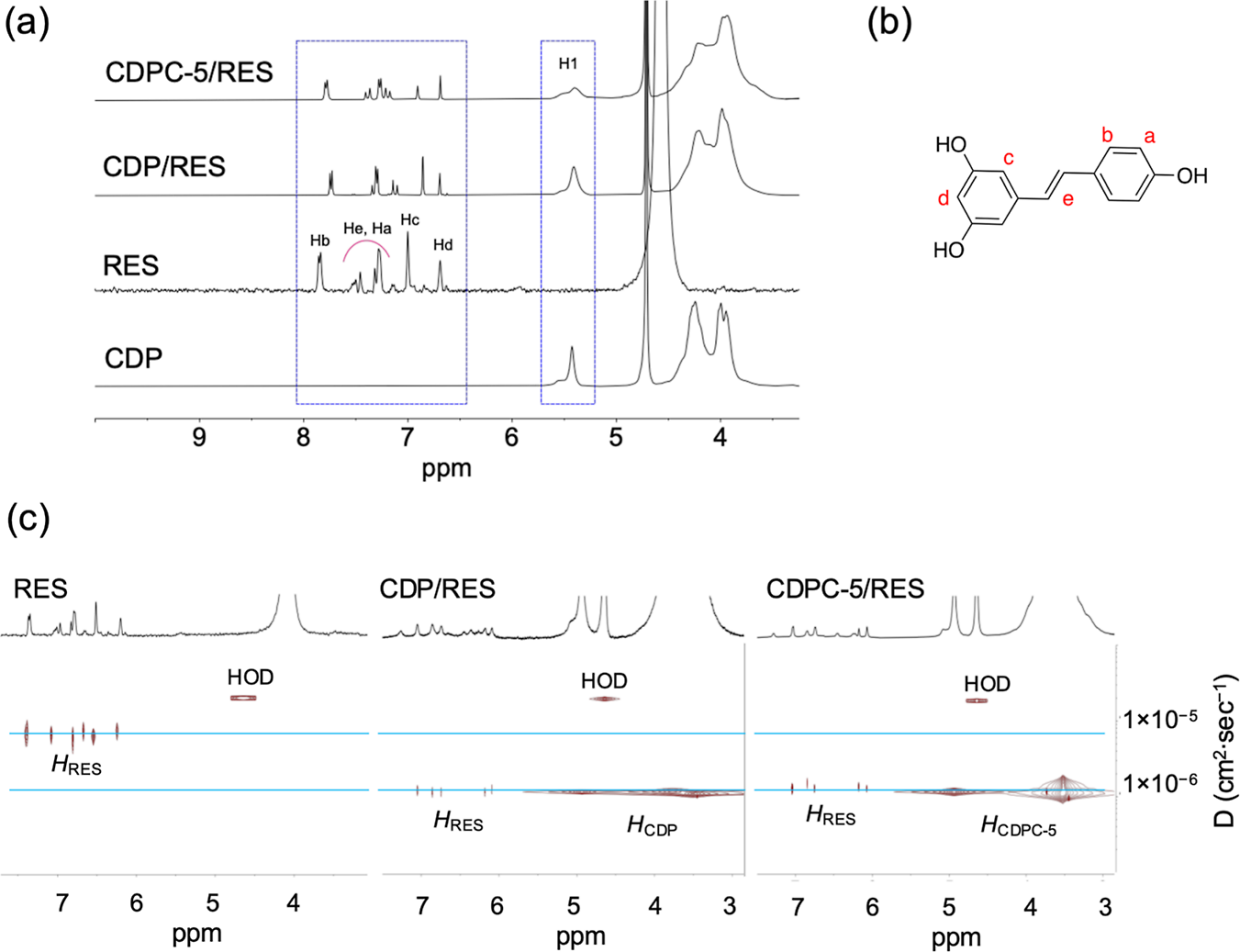
(a) ^1^H NMR spectra (600 MHz, 25 °C) of RES, CDP, CDP/RES, and CDPC-5/RES in D_2_O. (b) The chemical structure of RES is provided to assist in the spectral interpretation. (c) DOSY spectra (D_2_O, 600 MHz, 25 °C) of RES, CDP/RES, and CDPC-5/RES. The *x*-axis represents the ^1^H chemical shift of the samples in D_2_O, while the *y*-axis corresponds to the diffusion dimension. The ^1^H signal assignments are included in the spectra.

The integration of the anomeric proton signal of β-CD was used to determine the EC of the polymers by comparing it with the characteristic proton signals of RES in the ^1^H NMR spectrum. For CDP/RES, the integration ratio of β-CD to RES was found to be 1:0.6. Considering that the β-CD content in CDP was 72% (as determined by ^1^H NMR), the calculated EC was 0.09 g of RES per 1 g of CDP. Similarly, for CDPC-5/RES, the integration ratio of β-CD to RES was 1:0.9, and with a β-CD content of 55%, the calculated EC was 0.10 g of RES per 1 g of CDPC. Both CDP and CDPC exhibited comparable EC values as determined by ^1^H NMR spectroscopy, which is consistent with the results obtained from UV–vis spectroscopy. Although a discrepancy of ±0.09 g was observed between the EC values derived from ^1^H NMR and UV–vis measurements, this variation is considered acceptable.

Importantly, these data also show that despite the lower β-CD content in CDPC relative to CDP, which would typically result in a lower EC, similar EC values are observed for both polymers (Figure 3b). This further supports the data shown in Figure 3a,b, which indicate that the incorporation of CyG enhances the polymer/RES interactions that compensate for the reduced β-CD content in CDPC compared with CDP. CyG facilitates inclusion complexation with β-CD, effectively increasing the ratio of RES to available β-CD in CDPC relative to CDP by approximately 50%.

As noted in Section 3.3, DOSY NMR is a powerful technique for mixture analysis^47,48^ enabling the separation of chemical species based on their diffusion coefficients. Beyond the mixture characterization, DOSY NMR is employed to study host–guest interactions between cyclodextrins and guest molecules.^9^ For instance, the complexation of vanillin with CD-based polymer has been demonstrated by a decrease in the self-diffusion coefficient of vanillin upon forming a complex with the larger, slower-diffusing polymeric host (CD-based polymer).^9^ In our work, DOSY NMR analysis was performed to investigate the interaction of RES with CDP and CDPC-5. Figure 4c presents the DOSY spectra for RES, CDP/RES, and CDPC-5/RES in D_2_O. The diffusion coefficient of free RES was significantly higher than that of RES encapsulated within CDP and CDPC-5, indicating restricted molecular mobility upon complexation. In both CDP/RES and CDPC-5/RES samples, the diffusion coefficient of RES closely matched that of the corresponding polymer species, suggesting that RES molecules diffuse together with the polymer matrix, being a part of a stable host–guest complex. A comparison between the CDP and CDPC-5 revealed no difference in RES diffusion behavior, implying that both polymers exhibit comparable inclusion efficiency. These findings align with a previous report that decreased guest diffusivity and matched diffusion coefficients between host and guest are reliable indicators of effective encapsulation within cyclodextrin-based polymeric systems.^9^

#### 3.5.2 DLS and Zeta Potential

Figure 5 shows DLS data for CDP and CDPC-5, each at a concentration of 5 mg·mL^–1^ in saline, as well as their complexes CDP/RES and CDPC-5/RES at concentrations of 5 mg·mL^–1^ and 10 mg·mL^–1^ in saline. The results include both number and volume distributions to assess the hydrodynamic size and dispersity of the particles. For CDP and CDP/RES, these distributions reveal a consistent average size around 3.5 ± 0.6 nm, indicating a monodisperse population for both concentration levels. This value is also consistent with previously reported hydrodynamic sizes for CDP.^3,9^ In contrast, CDPC-5 and CDPC-5/RES show larger hydrodynamic sizes, with number-based distributions of approximately 13 ± 3 nm and volume-based distributions increasing to 19 ± 7 nm. Despite the slightly increased size and dispersity in CDPC-5, the DLS profiles in Figure 5 still suggest a generally monodisperse system.

**Figure 5:**
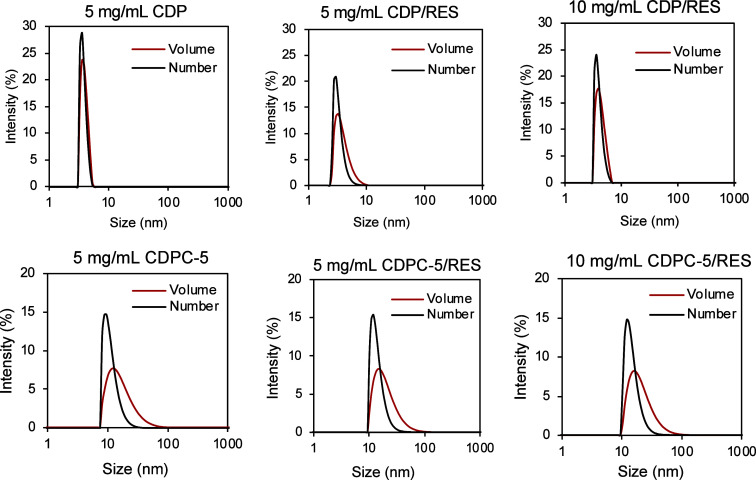
DLS data for CDP and CDPC-5 at a concentration of 5 mg·mL^–1^ in saline and their complexes with RES at concentrations of 5 mg·mL^–1^ and 10 mg·mL^–1^ in saline. Data are reported in both volume and number distributions.

The broader volume distributions relative to the number distributions for CDPC-5 suggest the presence of a minor population of larger particles or aggregates. This can be attributed to the presence of CyG groups in the CDPC-5 structure, which likely contributes to increased intermolecular interactions. Importantly, the hydrodynamic size and size distribution remained relatively stable upon complexation with RES for both CDP and CDPC-5 at both concentrations, indicating that particle formation is predominantly governed by the intrinsic properties of the polymer network rather than by the presence of the guest molecule.

Figure S2 presents the zeta potential measurements for CDP and CDPC-5, both at a concentration of 5 mg·mL^–1^ in saline, as well as their respective complexes CDP/RES and CDPC-5/RES at concentrations of 5 mg·mL^–1^ and 10 mg·mL^–1^ in saline. All samples exhibited zeta potential values within a slightly negative range, approaching neutrality, with no significant variation observed based on the concentration of the complexes or the presence of RES. It is important to note that nanoparticles with a zeta potential between −10 mV and +10 mV are typically considered as approximately neutral,^56^ which is consistent with the values observed in all samples analyzed in this study. A key finding from these measurements is that the amine group of CyG remains nonprotonated at physiological pH, attributable to its weakly basic nature, with a p*K*_b_ value of 14.4.^57^ Consequently, CyG exists in a neutral state under physiological conditions while maintaining the ability to engage in hydrogen bonding and dipolar interactions within polymeric and biological environments. This characteristic is significant in the context of cytotoxicity, as the cationic charge is typically responsible for cytotoxicity worsening.^23,58^

### 3.6 ABTS Antioxidant Activity

The ABTS antioxidant activity assay is based on the ability of antioxidants to neutralize the ABTS^•+^ radical cation, a stable and intensely colored blue-green radical generated by the oxidation of ABTS. Upon reaction with antioxidants, the ABTS^•+^ radical is reduced, leading to a decrease in absorbance at a characteristic wavelength of 734 nm. The extent of this reduction is proportional to the antioxidant capacity of the sample, providing a quantitative measure of its free radical-scavenging ability.

Figure 6 represents the reduction of ABTS^•+^ by β-CD, CDP, and CDPC-5 in the concentration range between 0.12 and 0.48 mg·mL^–1^ after 15 min of reaction. β-CD exhibits low ABTS^•+^ scavenging ability, achieving a reduction of only about 4% at a 0.24 mg·mL^–1^ concentration. CDP exhibits slightly enhanced activity to 7% at 0.24 mg·mL^–1^ and 22% at 0.48 mg·mL^–1^. Interestingly, CDPC-5 provides a substantially greater antioxidant effect than CDP, reaching 14, 53, and 72% reduction upon increasing its concentration from 0.12 to 0.48 mg·mL^–1^. To the best of our knowledge, there is currently no evidence in the literature reporting CyG as an effective radical scavenger or antioxidant. Therefore, our findings suggest that the incorporation of CyG into polymeric structures, containing ether and hydroxyl functionalities with inherently low radical-scavenging capacity, markedly improves the antioxidant activity of resulting polymers. Furthermore, the antioxidant activity of CDP and CDPC with encapsulated RES was assayed. At a concentration of 0.12 mg·mL^–1^, the antioxidant activity for both CDP/RES and CDPC/RES complexes reaches a level of around 90%, i.e., much higher than in the absence of RES. This result is not surprising as RES is known for its strong radical-scavenging activity.^40^

**Figure 6:**
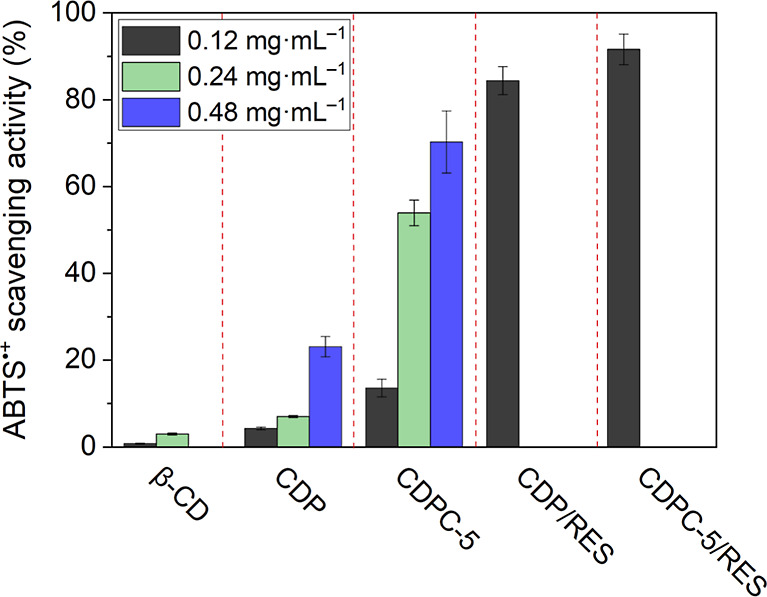
ABTS^•+^ scavenging activity expressed in % for β-CD, CDP, and CDPC-5, with different concentrations from 0.12 to 0.48 mg·mL^–1^, and for CDP/RES and CDPC-5/RES with concentrations of 0.12 mg·mL^–1^.

### 3.7 Metabolic Activity by the XTT Assay

The XTT assay was performed to assess the metabolic activity of human chondrocytes in the presence of CDP and CDPC polymers, both with and without RES. Figure 7 illustrates the trends in metabolic activity as polymer concentrations increased from 0.125 to 10 mg·mL^–1^ over 24, 48, and 72 h. The results indicate a progressive decline in metabolic activity with increasing CDP concentrations, suggesting a potential cytotoxic effect at higher doses. In contrast, no such decline was observed for CDPC polymers across the tested concentration range, highlighting a distinct difference between CDP and CDPC. This enhancement in cellular viability may be attributed to the presence of CyG moieties, which possess the capacity to form hydrogen bonding interactions with cellular biomolecules, potentially enhancing cellular function via the cell–polymer interactions. Similar trends have been observed in zwitterionic guanidine-based polymers, which were found to mimic cell-penetrating peptides and enhance cellular uptake while maintaining low cytotoxicity.^58^ Furthermore, the intrinsic antioxidant activity of CDPC may contribute to chondrocyte protection against oxidative stress by neutralizing reactive oxygen species, a factor known to impair chondrocyte viability and function.^59^ The time-dependent variation is evident for all tested samples. At 24 h, the metabolic activity is lower than that of the control for all the samples. However, the recovery trends are observed at 48 and 72 h, particularly at 0.125 mg·mL^–1^, where the metabolic activity exceeds that of the control. At concentrations above 0.125 mg·mL^–1^, the metabolic activity increases after 48 h but declines after 72 h. These outcomes align with expectations, as chondrocyte phenotypic loss is known to occur in a 2D culture environment.^60^

**Figure 7:**
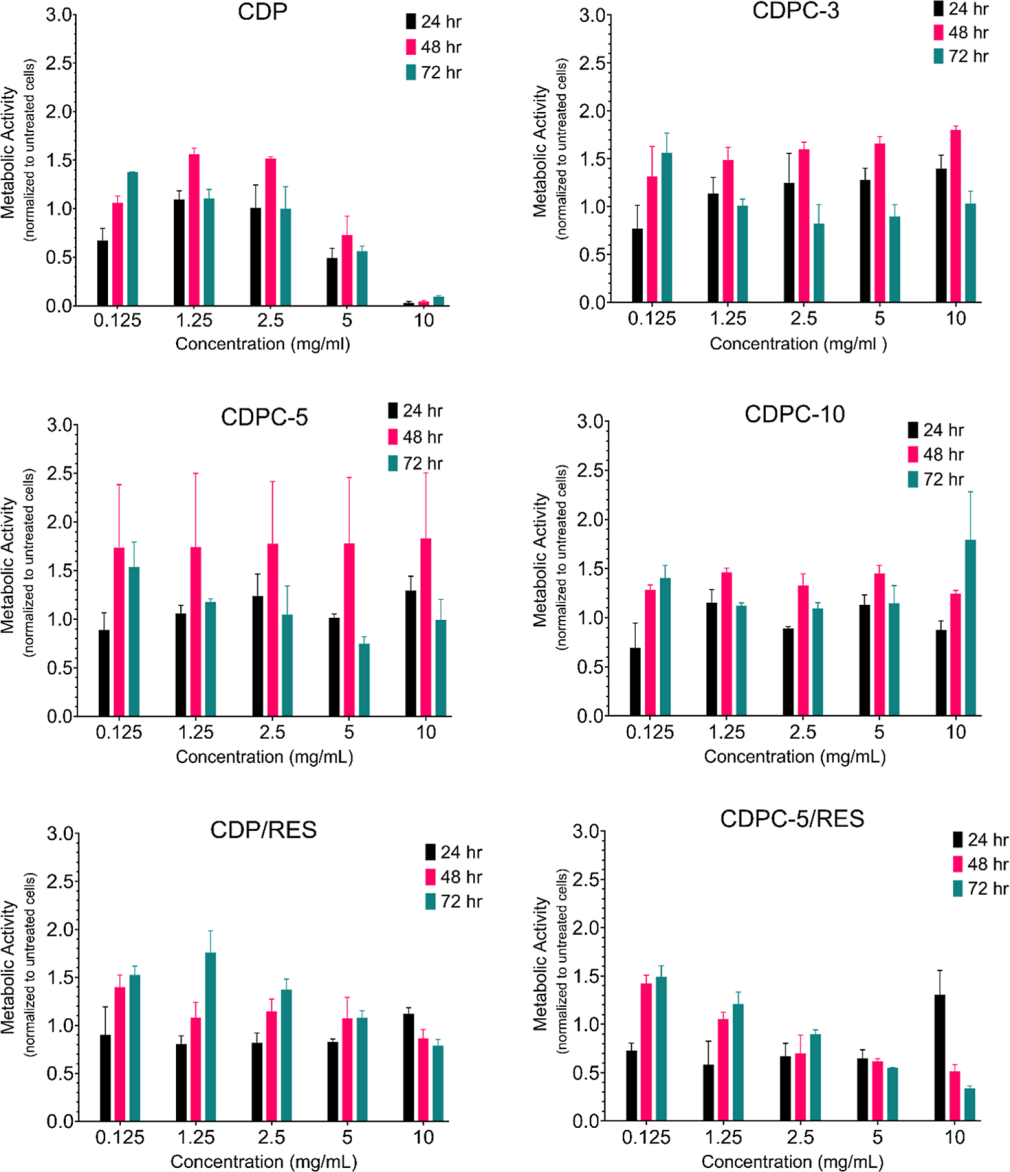
Metabolic activity (XTT assay) of human chondrocytes at a density of 5 × 10^3^ cells·cm^–2^ treated with CDP, CDPC-3, -5, -10, CDP/RES, and CDPC-5/RES at concentrations of 0.125 to 10 mg·mL^–1^ over 24, 48, and 72 h. Data is normalized to untreated cells. Data are presented as mean ± SD (*n* = 3, chondrocytes from a single donor).

The metabolic activity of chondrocytes was also evaluated in the presence of RES-encapsulated polymers. For the CDP/RES complex, the metabolic activity is maintained even at higher concentrations of 5 and 10 mg·mL^–1^, in contrast to CDP alone. Furthermore, CDPC-5/RES exhibits an increase in metabolic activity within the range of 0.125–2.5 mg·mL^–1^, particularly between 48 and 72 h, a trend that was not observed for CDPC-5 alone. These findings suggest a positive effect of RES incorporation in polymers.

### 3.8 Polymer Uptake with Chondrocytes

Efficient intracellular uptake of drug carriers is critical for cartilage-targeted drug delivery, as it enhances local drug availability, prolongs intracellular retention, and improves therapeutic efficacy.^37,38^ Encapsulation within nanoscale carriers further overcomes the poor bioavailability typically observed with free drugs.^37,38^

In this study, cellular internalization of CDP and CDPC-5 was assessed by labeling the polymers with FITC and monitoring uptake using CLSM. As shown in Figure 8, both 3D and 2D images demonstrate successful internalization and spatial distribution of the polymers within the cytoplasm. Homogeneous fluorescence in z-stacked images indicates endocytotic uptake rather than surface adsorption, confirming efficient intracellular localization. These results highlight the effective cellular internalization of both CDP and CDPC-5, supporting their potential as intracellular drug delivery platforms.

**Figure 8:**
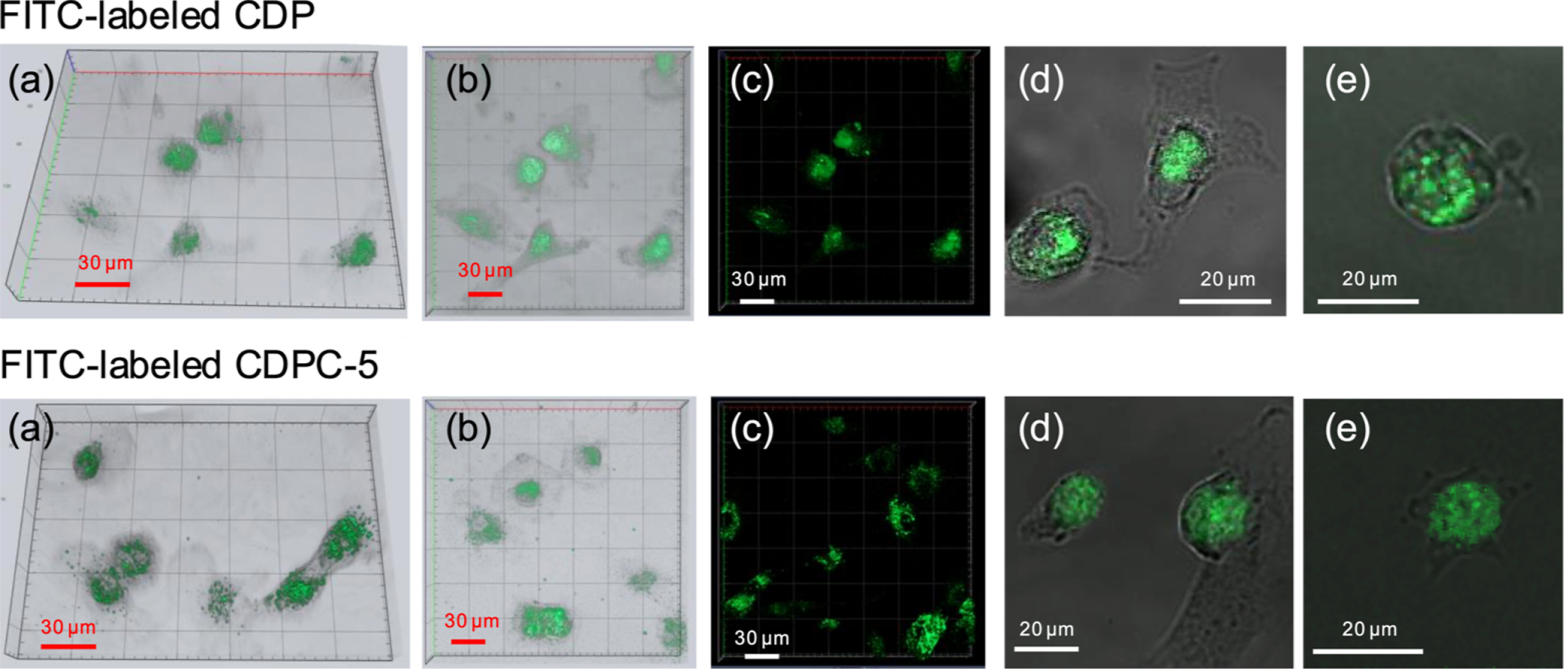
Confocal laser scanning microscopy (CLSM) images of the FITC-labeled CDP and CDPC-5 uptake by chondrocytes. 3D images in transparent (a,b) and standard (c) modes illustrate the spatial distribution of polymers within the cells, and 2D images (d,e) depict their intracellular localization.

The observed uptake is consistent with the physicochemical properties of both carriers. CDP and CDPC-5 have hydrodynamic sizes of ∼5 and ∼15 nm, respectively, and a near-neutral surface, which represent favorable conditions for chondrocyte internalization. For chondrocyte uptake, carriers with hydrodynamic sizes in the range of 10–100 nm have been reported.^61^ However, highly cationic particles can cause cytotoxicity or aggregation, whereas neutral or mildly anionic polymers, such as PEGylated carriers, enhance colloidal stability while still supporting effective cellular internalization.^62^ Lima et al. reported that negatively charged PEGylated micelles and liposomes (−25 to −27 mV) were internalized more efficiently by chondrocytes than cationic chitosan-hyaluronic acid NPs (+28 mV).^38^ The nanoscale dimensions and surface potential of CDP and CDPC-5 support their efficient uptake by chondrocytes, as confirmed by confocal microscopy. Once internalized, these carriers provide an effective platform for enhancing the intracellular delivery and therapeutic efficacy of RES, as further evaluated through cartilage matrix deposition.

### 3.9 Cartilage Matrix Production

Chondrogenic gene expression was performed on human chondrocytes obtained from two donors, labeled as donors I and II. The experiments were carried out with CDP/RES and CDPC-5/RES, with results presented in Figure 9. Some genes, which include COL2A1 for cartilage-specific ECM, ACAN for cartilage-specific proteoglycan major protein, SOX9 for a chondrogenic transcription factor, MMP13 and MMP3 involved in the remodeling of hypertrophic cartilage, and COL1A1 engaged in fibrous tissue formation, and GAPDH as a housekeeping gene, were analyzed.^63^ For ACAN, both CDP/RES and CDPC-5/RES enhance the level of this gene on day 3 in both donors compared to the control. In donor I, CDPC-5/RES provides a more remarkable fold change in SOX9 than CDP/RES. In donor II, CDPC-5/RES also has a significant effect, although the expression level is relatively low compared with donor I. CDP/RES shows a mild increase in the expression of both donors. The controls are also constant at the baseline level, and no changes are observed. The levels of COL2A1 also differ between the two donors. In donor I, CDPC-5/RES suppresses the expression of COL2A1 on day 3, while CDP/RES shows a recovery trend. In donor II, CDP/RES enhances the expression level of COL2A1 on day 3 even more than does CDPC-5/RES, which suppresses the expression level. Between the two donors, donor II always expresses lower levels of COL2A1 than donor I. Both treatments in this study suppress the expression of COL1A1 more than the untreated controls. CDPC-5/RES is more effective in downregulating COL1A1 in both donors, especially on day 3, with a higher level in donor I compared with donor II. CDP/RES also downregulates COL1A1 in a less efficient way than does CDPC-5/RES.

**Figure 9:**
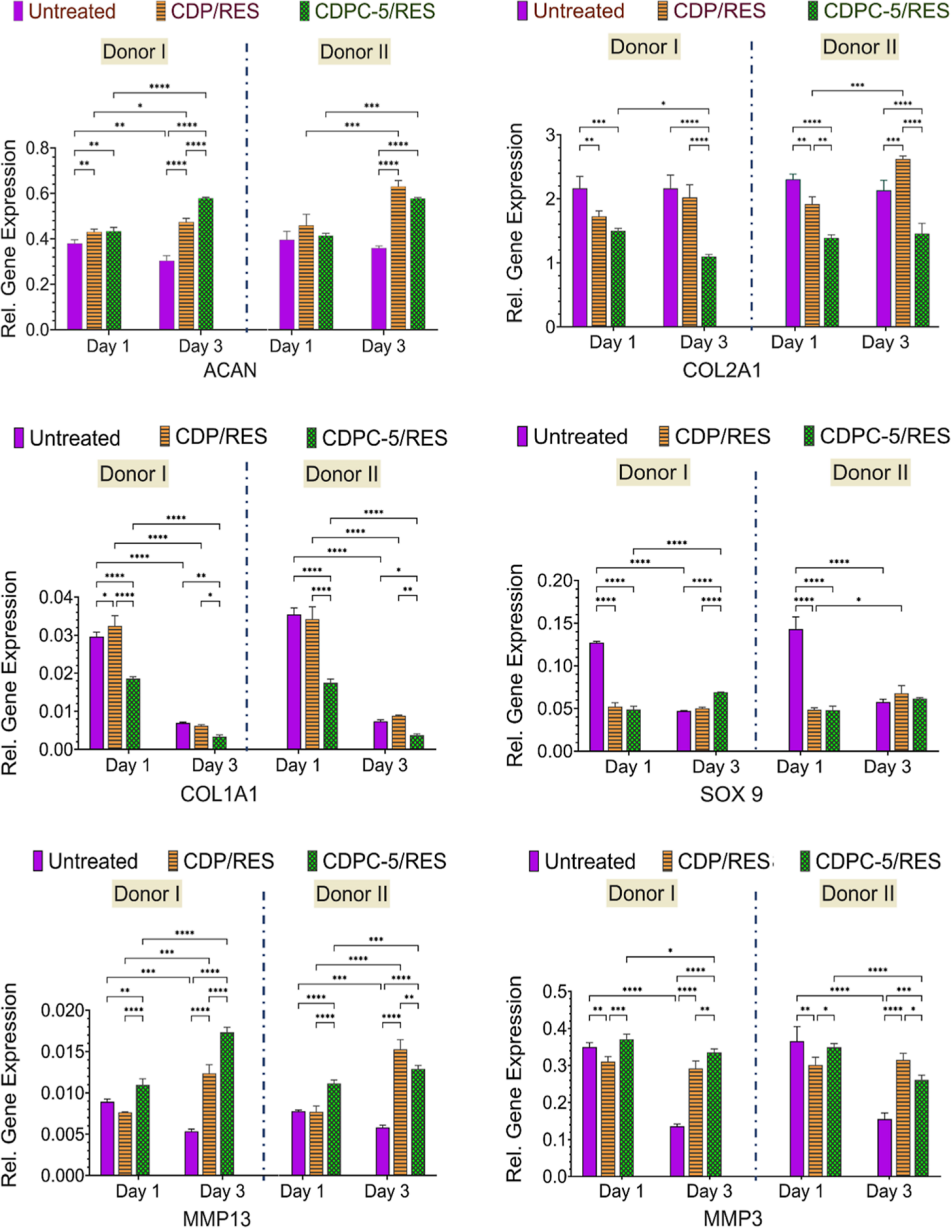
Gene expression profiles of cartilage-specific and degradation-related markers for two donors of chondrocytes (donor I and donor II). Relative gene expression levels of ACAN, COL2A1, SOX9, COL1A1, MMP13, and MMP3 were evaluated in chondrocytes treated with 0.125 mg·mL^–1^ CDP/RES and CDPC-5/RES for 1 and 3 days. Untreated samples are used as controls. Gene expression is normalized to housekeeping genes and is presented relative to untreated controls. Data are shown separately for donor I and donor II. All statistical analyses were performed using two-way ANOVA followed by Tukey’s post hoc test, using GraphPad Prism version 10. Data are presented as mean ± SD (*n* = 3). *p < 0.05, **p < 0.01, ***p < 0.001, ****p < 0.0001.

The controls are generally stable throughout the experiment, with no noticeable changes in the levels. SOX9 is found to be significantly expressed at a higher level by CDPC-5/RES in both donors by day 3. The level of SOX9 expression is also increased in both donors. Nonetheless, CDP/RES has a smaller impact than CDPC-5/RES.^64^ This upregulation by CDPC-5/RES suggests an increasing expression trend from day 1 to day 3, while CDP/RES shows a relatively constant increase over the same period. The catabolic enzymes MMP3 and MMP13 are overexpressed at higher levels by CDPC-5/RES in both donors than by CDP/RES and the untreated controls. In donor I, the expression of these enzymes is highest on day 3. Donor II also increases MMP13 expression with CDPC-5/RES, but the levels are much lower than in donor I. CDP/RES in this study expresses lower levels of MMP3 and MMP13 in both donors. The MMP3 and MMP13 levels are generally unchanged in the untreated controls and are typically low. This donor-dependent increase in MMP3 and MMP13 suggests that CDPC-5/RES may transiently activate remodeling pathways rather than exclusively promote degenerative signaling. Given that MMPs also participate in physiological matrix turnover, their elevation, particularly in donor I, may reflect an adaptive or early remodeling response to the formulation. Nonetheless, the magnitude of the increase indicates a formulation-specific effect that warrants consideration in future optimization.

The results of CDP/RES and CDPC-5/RES treatments on donor I and donor II chondrocytes suggest different therapeutic potentials compared to untreated cells for cartilage repair. A day 1 vs day 3 trends analysis demonstrates that CDPC-5/RES produced more robust effects by day 3, and there are more considerable fold changes for most of the genes analyzed. CDP/RES shows a steadier increase, while the untreated controls remain unchanged. Overall, CDPC-5/RES demonstrated superior efficacy in enhancing anabolic activity and chondrocyte differentiation, as indicated by significant upregulation of ACAN and SOX9 in both donors. However, the concurrent increase in MMP3 and MMP13 highlights the formulation-dependent balance between anabolic stimulation and matrix-remodeling responses, which may vary among donors and should be optimized to minimize unintended catabolic activity. CDP/RES demonstrates a reduced efficacy in enhancing anabolic markers but provides a more balanced strategy with a diminished risk of excessive catabolic activity, notably reflected in its capacity to maintain COL2A1 expression in donor II. These findings highlight the importance of tailoring treatments to individual donor responses, with CDPC-5/RES being ideal for robust matrix synthesis and differentiation and CDP/RES serving as a safer alternative for maintaining ECM stability. The follow-up in vivo data are expected to demonstrate the potential of the systems investigated in this work in cartilage repair.

## 4 Conclusions

In this study, a novel β-cyclodextrin–epichlorohydrin–cyanoguanidine (CDPC) polymer was synthesized as an advanced member of the β-cyclodextrin–epichlorohydrin (CDP) family. The incorporation of cyanoguanidine (CyG) introduces nitrogen-rich functional groups, enhancing the polymer’s potential for molecular interactions relevant to pharmaceutical and regenerative medicine applications. Comprehensive structural characterization confirmed the successful integration of CyG into the polymer network, with the molar ratios of [β-CD]_p_ to [CyG]_p_ ranging from 1:1.3 to 1:2.6. The resulting CDPC exhibited a lower β-CD content (55 wt %) compared to conventional CDP (72 wt %).

The incorporation of CyG provided the polymer network with nitrogen-containing functionalities characterized by a weak basicity (p*K*_b_ 14.4), resulting in a nonprotonated and neutral structure at physiological pH. These functionalities provide additional hydrogen bonding and dipole–dipole interaction sites without imparting a cationic charge, thereby enhancing the polymer interaction capacity. As a result, the CDPC polymer exhibited superior resveratrol (RES) complexation ability compared to CDP, despite its lower β-CD content. Phase-solubility and encapsulation studies revealed enhanced RES loading capacity and encapsulation efficiency, which can be attributed to the additional interaction sites provided by CyG. Moreover, CDPC exhibited intrinsic antioxidant activity, which was further enhanced upon RES encapsulation.

Physicochemical characterization indicated the formation of nanoscale RES–polymer complexes (hydrodynamic diameters 4 nm for CDP/RES and 18 nm for CDPC/RES) with neutral to slightly negative surface potentials, properties favorable for polymer uptake by chondrocytes. In vitro assays using human chondrocytes confirmed the cytocompatibility and efficient cellular internalization of FITC-labeled CDP and CDPC, as visualized by confocal laser scanning microscopy. Treatment with CDP/RES and CDPC/RES supported chondrocyte metabolic activity and uptake and modulated gene expression by enhancing key anabolic markers (ACAN, SOX9) and influencing matrix-associated genes in a donor-dependent manner, thus demonstrating the biological activity of these formulations and their potential relevance for cartilage repair. The observed variability among chondrocytes from different donors emphasizes the importance of considering patient-specific responses in future translational work.

Overall, this study highlights the potential of β-CD-based polymers, particularly CyG-functionalized CDPC, as safe and efficient drug carriers for cartilage repair. The incorporation of CyG provides a rational design approach to enhance drug–polymer interactions, antioxidant properties, and intracellular drug delivery, positioning CDPC as a promising platform for future cartilage regeneration therapies.

## Supplementary Material



## Data Availability

Supporting data associated with this article can be found online as a dataset at Zenodo: https://doi.org/10.5281/zenodo.19474927.

## Notes

FTIR spectra and zeta potential results (PDF)
