# Supplementary material for: Cyclodextrin–Epichlorohydrin–Cyanoguanidine Polymer for Resveratrol Delivery to Enhance Human Chondrocyte Function in Cartilage Repair
Source: Biomacromolecules. 2026 Apr 22;27(5):3063–79. doi: 10.1021/acs.biomac.5c02392 (PMC13169299; doi:10.1021/acs.biomac.5c02392)
Supplement: Supplementary file 1 [file bm5c02392_si_001.pdf]

Cyclodextrin-epichlorohydrin-cyanoguanidine polymer for resveratrol delivery to enhance human chondrocyte function in cartilage repair

Mahmoud A. Elmeligy,[a, ‡] Forough Rasoulian,[b, ‡] Shima Kalantarifard,[a] Juraj Filo,[c] Sahar Dinparvar,[d] Ahmed M. Omer,[a] Lucy Vojtová,[d] Stefan Nehrer,[b] Igor Lacík,[a,] Abolfazl Heydari[a,d,*]*

[a]Polymer Institute of the Slovak Academy of Sciences, Dúbravská cesta 9, 845 41, Bratislava, Slovakia

[b]Center for Regenerative Medicine, University of Continuing Education Krems, 3500 Krems, Austria

[c]Department of Organic Chemistry, Faculty of Natural Sciences, Comenius University, Ilkovičova 6, 842 15 Bratislava, Slovakia

[d]Central European Institute of Technology, Brno University of Technology, 612 00 Brno, Czech Republic

*Corresponding authors: abolfazl.heydari@savba.sk, igor.lacik@savba.sk

FIGURES

Figure S1. FT-IR spectra of polymers

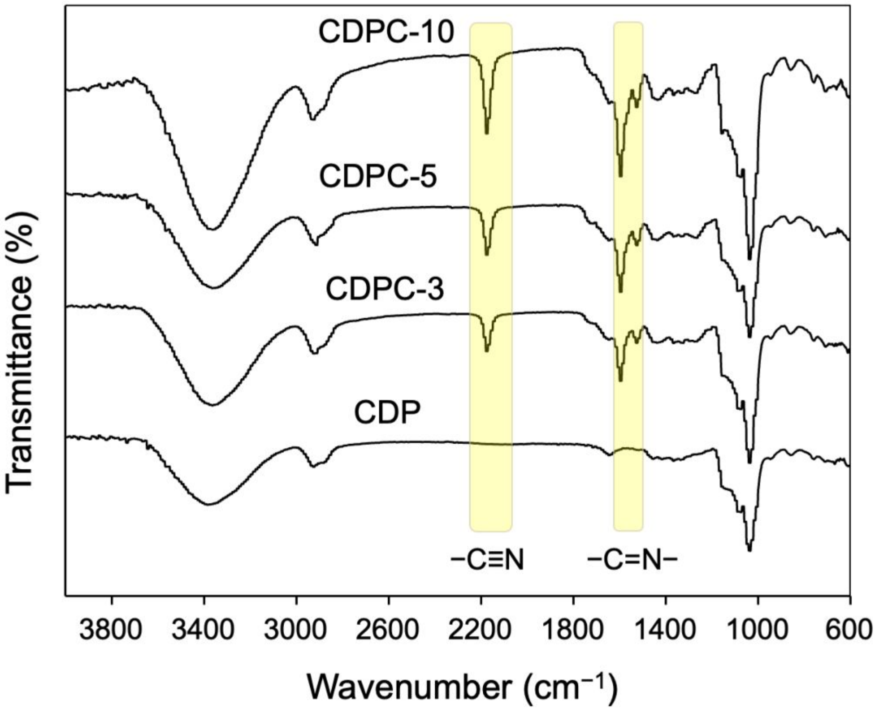

Figure S1. FT-IR spectra of CDP, CDPC-3, -5, and -10. The signals related to cyano (–C≡N) and –C=N– groups are highlighted in yellow.

Figure S2. Zeta potential of polymers and their complexes with RES

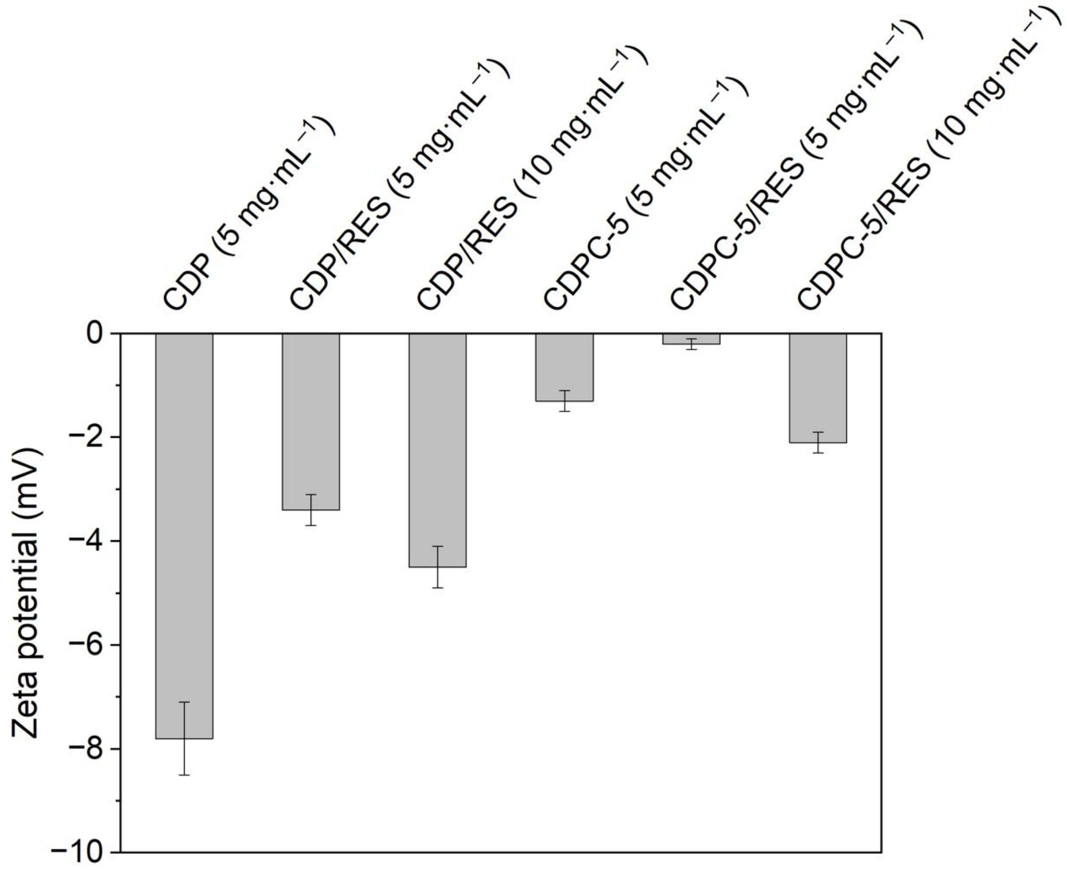

Figure S2. Zeta potential of CDP and CDPC-5 at a concentration of 5 mg·mL^{-1} in saline, and their complexes with RES at concentrations of 5 mg·mL^{-1} and 10 mg·mL^{-1} in saline.
